# Multiplexed Detection of Reactive Biomolecules via Chemoresponsive DNA Accumulation on Fluorescence-Encoded Beads

**DOI:** 10.1021/cbmi.5c00226

**Published:** 2026-01-08

**Authors:** Tatsuya Nishihara, Masato Sugawara, Reoto Mio, Yuto Motohashi, Kazuhito Tanabe

**Affiliations:** Department of Chemistry and Biological Science, College of Science and Engineering, Aoyama Gakuin University, 5-10-1 Fuchinobe, Chuo-ku, Sagamihara, Kanagawa 252-5258, Japan

**Keywords:** Beads accumulation, Probe DNA, Fluorescent sensor, H_2_O_2_, Nitroreductase, Multiplex analysis

## Abstract

The multiplex analysis of reactive biomolecules is crucial in diagnostics and life science research. However, conventional methods using small-molecule-based fluorescent probes are limited in the number of simultaneously detectable targets because of the spectral overlap of their fluorescence wavelengths. Herein we present a sequencing-free multiplexed analysis platform using chemoresponsive DNA-based fluorescent probes. A target-responsive moiety was installed in the phosphate backbone of the probe to strategically destabilize the DNA duplex. Reaction with a target molecule such as hydrogen peroxide or nitroreductase cleaved this moiety, restoring duplex stability and thereby triggering the accumulation of fluorescent product DNAs on beads functionalized with a complementary sequence. By encoding beads with distinct fluorescence intensity ratios and sizes, we achieved the simultaneous and specific detection of multiple targets in a single sample. The system performed well even with complex biological samples. This modular “reaction-to-accumulation” strategy offers a generalizable approach for developing DNA-based multiplex detection systems tailored to different target molecules.

## Graphical abstract

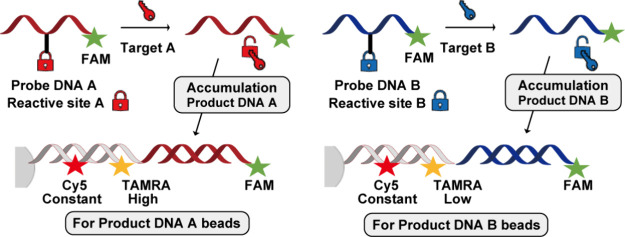

## Introduction

In living organisms, various reactive oxygen species (ROS) and enzymes interact to sustain cellular functions and homeostasis through intricate processes. Different ROS, including superoxide, hydrogen peroxide (H_2_O_2_), and hydroxyl radicals, are produced and eliminated under a tightly controlled balanced mechanism,^1^ whereas various enzymes, such as proteases and oxidoreductases, regulate intracellular metabolism and signal transduction.^2^ Disturbances in ROS homeostasis and enzyme activity have been associated with a range of pathological conditions. In this context, the multiplex detection of reactive biomolecules at the molecular level is essential for the accurate elucidation of disease mechanisms and therapeutic targets.

Recent developments in fluorescent probe technology have enabled the real-time and highly sensitive detection of enzyme activities and individual ROS, greatly advancing the analysis of molecular dynamics at the cellular, tissue, and organismal levels.^3^ Fluorescent probes that selectively react with specific enzymes or ROS to generate visible fluorescent signals have become powerful tools for elucidating molecular networks, understanding disease mechanisms, and developing diagnostic and therapeutic strategies. Despite these advances, comprehensive multiplexed analysis of ROS and enzymatic activities remains limited by the spectral overlap of the fluorescence wavelengths. This color barrier (4–6 components) constrains the number of targets that can be detected simultaneously. Furthermore, understanding the intrinsic chemical diversity of these reactive biomolecules and their roles in complex diseases requires technologies capable of simultaneously detecting multiple reactive species.

Multiplex strategies incorporating Raman spectroscopy and DNA-based approaches have enabled the simultaneous detection of diverse reactive biomolecules. Raman spectroscopy leverages the unique vibrational spectra of each molecule, allowing simultaneous detection of numerous target molecules without the interference of spectral overlap.^4,5^ Indeed, Raman methods have demonstrated good performance in simultaneous detection of multiple reactive biomolecules such as enzymes with high selectivity.^6–8^ However, these approaches require specialized and high-cost instrumentation, thus limiting their versatility compared to conventional fluorescence analysis. In contrast, DNA-based approaches have emerged as powerful tools for biosensing. For instance, aptamers obtained via Systematic Evolution of Ligands by Exponential Enrichment (SELEX)^9,10^ utilize DNA’s ability to fold into specific structures for high-affinity target recognition. Beyond such affinity-based detection, DNA can also be designed to read and quantify DNA sequences through reactions with reactive molecules.^11–14^ However, most of these DNA detection methods require next-generation sequencing (NGS). While NGS provides exceptionally high multiplexing capabilities (discriminating over 10 biomolecules) and comprehensive coverage,^15,16^ these technologies are limited by costs, processing time, and specialized infrastructure.

To achieve a sequencing-free readout, we focused on a decoding strategy that relied on fluorescence signals. Several approaches have been proposed to decode DNA sequence information by analyzing fluorescence signals^17–20^ or by leveraging not only fluorescence intensity but also the spatial organization of fluorophores within DNA nanostructures.^21–25^ In this study, we introduce a modular platform based on chemoresponsive DNA oligomers labeled by fluorescent molecules (Probe DNAs) and fluorescence-encoded beads (Figure 1). In the present system, the DNA generated in response to a chemical reaction with a target molecule selectively accumulates on fluorescently encoded beads bearing complementary sequences. The fluorescence intensity derived from the beads (TAMRA and Cy5) and the resulting FAM-labeled DNA product (Product DNA) enable the quantitative and simultaneous detection of multiple target molecules. This approach avoids cumbersome sequencing processes and offers high-throughput detection capabilities for the detection of reactive biomolecules. By decoding accumulated Product DNA using the fluorescence ratio (TAMRA/Cy5 ratio) and the size of each bead, we can overcome the fundamental limitations of spectral overlap.

**Figure 1:**
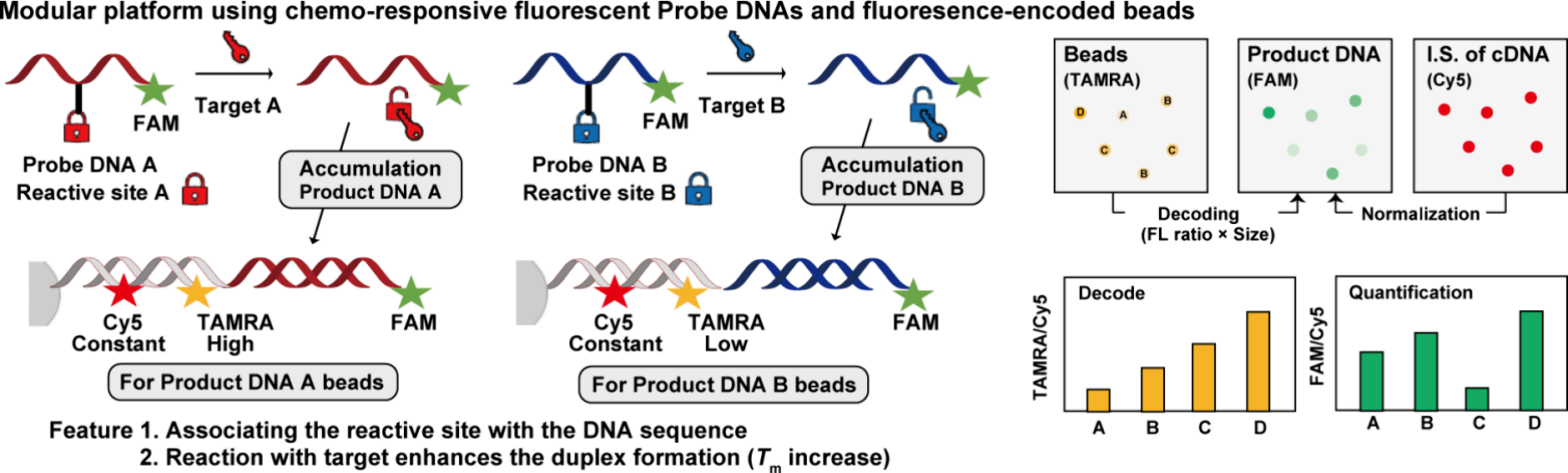
Schematic illustration of simultaneous detection of reactive biomolecules using chemoresponsive fluorescent Probe DNAs and fluorescent encoded beads.

## Results and Discussion

The Probe DNAs designed in this study were prepared by introducing reactive sites into the phosphate backbone of DNA and modifying the termini with the fluorescent dye FAM (Figure 1). These Probe DNAs had two important features: First, high sequence specificity was maintained by associating the reactive site with the DNA sequence. Second, upon reaction with the target molecule, the reactive site was cleaved, leading to enhancement of double-strand formation ability. Consequently, only the Product DNA generated upon reaction with the target molecule selectively accumulated on beads labeled with complementary sequences.

In this study, hydrogen peroxide, a representative ROS,^26^ and nitroreductase, a cancer-related enzyme,^27^ were selected as the target molecules. To introduce specific reactive sites for the targets, 4-(boronic acid pinacol ester)-substituted benzyl bromide or *p*-nitrobenzyl bromide was coupled with a thiophosphate group in the DNA strand.^28^ The reactions of these benzyl bromides with thiophosphate groups proceeded quantitatively (Figure S1). The reaction with each target (H_2_O_2_ or nitroreductase) led to transformation of the reactive site into a phenol or aniline structure, followed by self-immolation to be removed from the DNA (Scheme 1).^28^

**Scheme 1:**
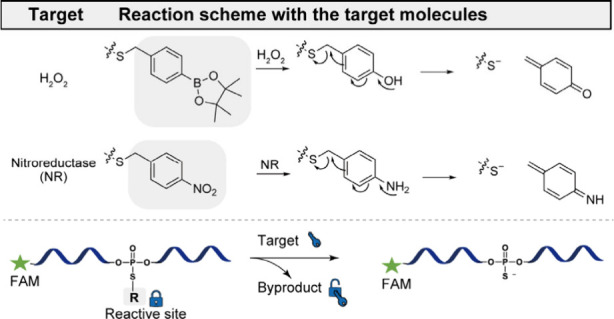
Chemical Conversion Depending on the Reactions with the Targets (H_2_O_2_ and Nitroreductase) in This Study

The effects of the position and number of reactive sites on the strand for hybridization were systematically evaluated (Figure 2). We measured the melting temperature (*T*_m_) of double-stranded DNA consisting of Probe DNAs or Product DNAs (as a model strand of reaction product) in the presence of their complementary strands to analyze their stability for duplex formation. The introduction of reactive sites in the central region resulted in a large decrease in *T*_m_. Furthermore, varying the number of modifications revealed that the introduction of three consecutive reactive sites significantly lowered *T*_m_ by over 10 °C. These findings suggest that the introduction of multiple reactive sites into the central region in 15-mer DNA could greatly alter *T*_m_, providing a useful probe design strategy (FAM-PB-DNA and FAM-NB-DNA). The significant decrease in *T*_m_ caused by the modification is strategically designed to prevent the nonspecific capture of unreacted probes. This thermodynamic instability is crucial for minimizing background signals, ensuring that only the reacted Product DNA, which possesses restored duplex stability, is captured by the beads.

**Figure 2:**
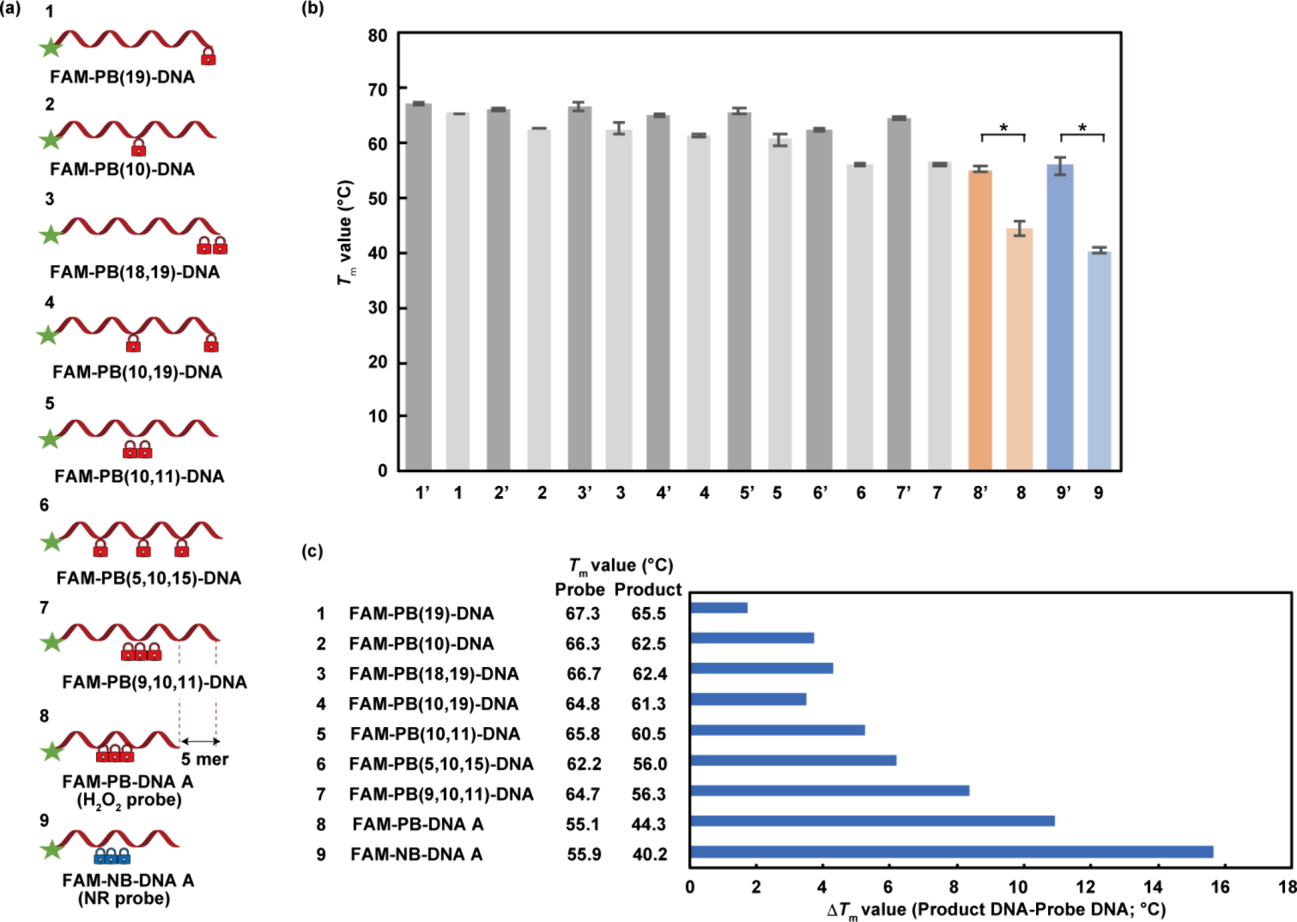
Change in the melting temperature (Δ*T*_m_) upon Product DNA formation. (a) Schematics of the DNA probe candidates. The padlock icon indicates the site of thiophosphate modification, and its color distinguishes the reactive moiety (red, PB moiety; blue, NB moiety). (b) Melting temperature (*T*_m_) analysis of Probe DNA and Product DNA. Numbers (e.g., 1) indicate Probe DNAs, and numbers with a prime (e.g., 1′) indicate the corresponding Product DNAs. Data are reported as mean ± SD (*n* = 3 for FAM-PB-DNA A, FAM-NB-DNA A, and its product; *n* = 2 for others). Statistical significance was evaluated using Student’s *t* test (*p < 0.05). (c) Measured *T*_m_ values and the resulting Δ*T*_m_, which was calculated as *T*_m_(Probe) – *T*_m_(Product). *T*_m_ evaluation was performed with 4 μM Probe DNA/Product DNA and complementary DNA in 100 mM NaCl.

We next evaluated the double-strand formation ability of the beads labeled with complementary strands (Figure S2). In this system, it is critical that the unreacted Probe DNA is not captured by the beads, while the beads can capture only the Product DNA; thus, we compared the amount of DNA that was captured by the beads under different temperature conditions (4 °C, room temperature, and 37 °C). At 37 °C, there was almost no background accumulation of unreacted Probe DNAs on the beads, and only the Product DNAs accumulated. These results indicate that the temperature is a key determinant for the selective capturing of Product DNA on the beads. In addition, we found fluorescence emission of FAM-labeled Product DNA from the beads in a concentration-dependent manner (Figure S2c).

The reaction with each target (H_2_O_2_ or nitroreductase) led to the detachment of their corresponding reaction site from the Probe DNAs (Scheme 1). We tracked these reactions by high-performance liquid chromatography (HPLC) and confirmed that both Probe DNAs generated the Product DNAs in the presence of their targets (Figure S3). Regarding concentration dependence, intermediate products were observed at low target concentrations (H_2_O_2_ or nitroreductase), whereas further reactions produced more of the main product. In addition, we evaluated the specificity of the H_2_O_2_-responsive DNA probe against other reactive species using HPLC (Figure S4). The probe exhibited a negligible reaction with nitric oxide or peroxyl radicals, confirming its selectivity. While superoxide treatment yielded the product likely due to dismutation-derived H_2_O_2_ and hypochlorite caused nonspecific degradation, the probe demonstrated high selectivity, ensuring its utility in biological contexts. We also examined the target-dependent capture of the Product DNA by the beads. The FAM-labeled Probe DNA was treated with their targets and then mixed with beads labeled by their complementary strand. As shown in Figure 3, bright fluorescence of FAM from the beads was only observed when processed with the target. We confirmed that the fluorescence intensity was comparable to that of a model strand with the same sequence and structure as the Product DNA. These results strongly indicate that efficient chemical conversion and accumulation were achieved. Thus, the Product DNA selectively accumulated only in the presence of each reactive biomolecule (H_2_O_2_ or nitroreductase). To evaluate the quantitative capability of the platform, we examined the correlation between the target concentration (H_2_O_2_ and nitroreductase) and the bead-associated fluorescence. As shown in Figure S5, the fluorescence signal increased in a concentration-dependent manner, although saturation was observed at higher concentrations. This result demonstrates that the platform enables quantitative analysis based on the signal intensity.

**Figure 3:**
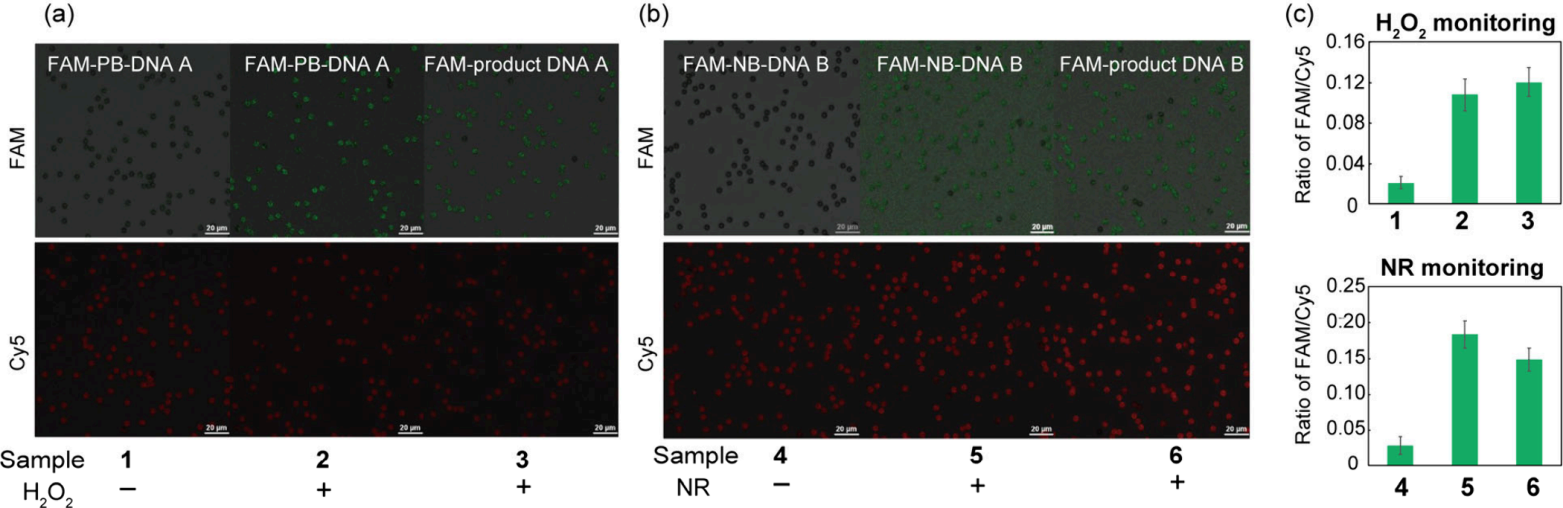
Target detection using FAM-PB-DNA for H_2_O_2_ and FAM-NB-DNA for nitroreductase. (a) Fluorescent images of bead from FAM and Cy5. 250 nM FAM-PB-DNA A was allowed to react with 0 or 1 mM H_2_O_2_ in 250 mM phosphate buffer (pH 7.4) for 1 h at room temperature. (b) Fluorescent images of bead from FAM and Cy5. 250 nM FAM-NB-DNA B was allowed to react with 0 or 500 μg/mL NR and 0 or 1 mM NADH in 250 mM phosphate buffer (pH 7.4) for 1 h at room temperature. After washing the beads solution, the reaction solutions were incubated with the cDNA labeled beads for 1 h at 37 °C to accumulate on the beads. (c) Fluorescence intensity ratio (FAM/Cy5) obtained from the beads in (a) and (b). Data are reported as mean ± SD (*n* = 30). Scale bars: 20 μm.

To enable multiplex detection, we prepared a panel of beads designed to be distinguishable from one another. We verified that spectral overlap among the fluorescent dyes (FAM, TAMRA, and Cy5) does not affect the quantification. As shown in Figure S6, the fluorescence intensities of beads in single-color controls were comparable to those in multicolor samples, confirming that crosstalk between channels is negligible under our optical conditions. We varied two parameters: the TAMRA/Cy5 fluorescence intensity ratio and the bead size (Figure S7). The TAMRA/Cy5 fluorescence intensity ratio for each bead was controlled by adjusting the amount of fluorescent label. This two-parameter encoding strategy allowed us to prepare a large number of beads with unique fluorescence–size signatures. Theoretically, the use of *N* fluorescence intensity patterns and *M* bead sizes enables *N* × *M* multiplex detections. The present system showed potential for at least 12 (4 fluorescence intensity ratios × 3 bead sizes) simultaneous detections under our experimental conditions (Figure S7).

Finally, we validated the multiplex detection of the targets (H_2_O_2_ and nitroreductase) using Probe DNAs that corresponded to each target and beads labeled with their complementary strands. The fluorescent intensity ratio of TAMRA and Cy5 (TAMRA/Cy5) on the beads enabled the identification of which DNA strand was captured by each bead (Figure S8). To assess specificity and multiplex performance, the reaction was performed under four conditions shown below. A cocktail of Probe DNAs (FAM-labeled PB-DNA A for H_2_O_2_, FAM-labeled NB-DNA B for nitroreductase) was incubated with different combinations of the targets: in the absence of both targets, with each target individually, or with both targets simultaneously. The resulting Product DNAs were then captured by the beads. Upon addition of H_2_O_2_, FAM fluorescence increased on high-intensity beads (TAMRA/Cy5 high), whereas addition of nitroreductase caused FAM accumulation on low-intensity beads (TAMRA/Cy5 low), confirming reaction-dependent accumulation and sequence-specific duplex formation, consistent with the design principle (Figure S9). Similar results were obtained even in the presence of 10% fetal bovine serum (Figure 4), demonstrating the applicability of this method to real biological samples.

**Figure 4:**
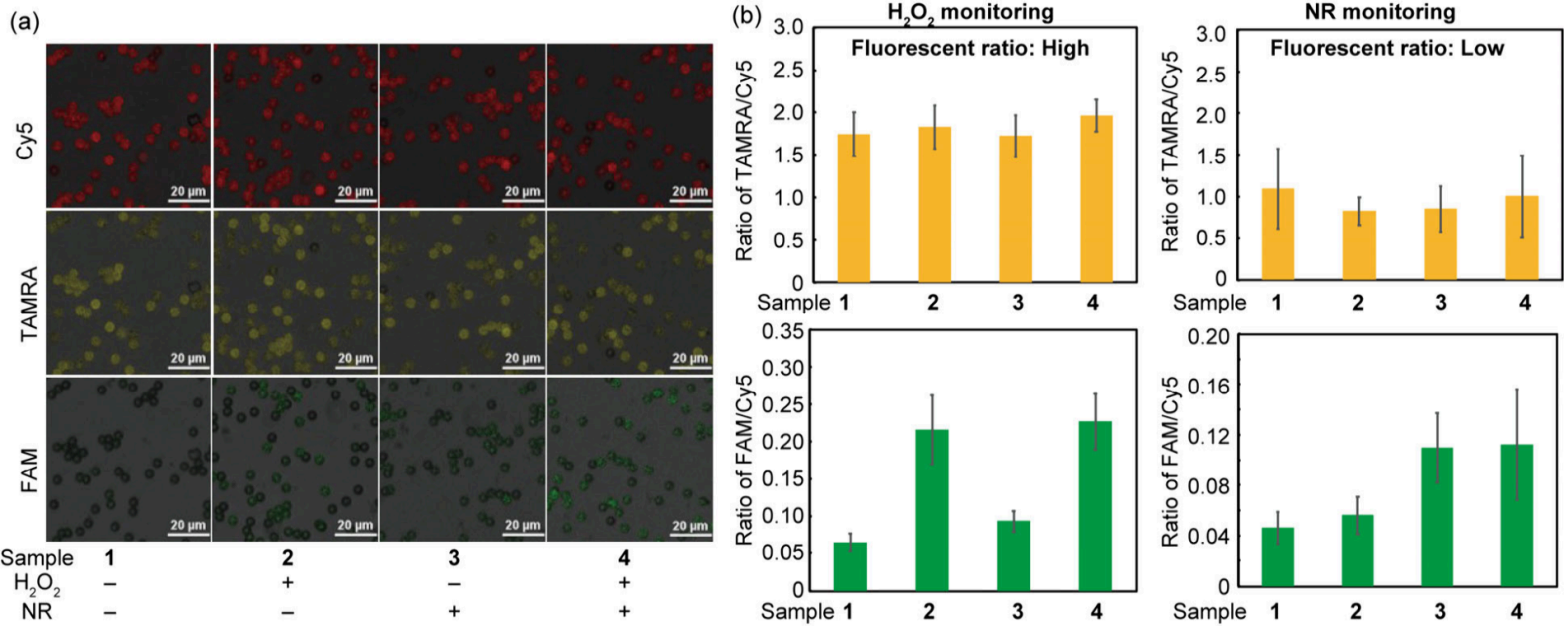
Simultaneous detection of H_2_O_2_ and nitroreductase as reactive biological molecules in bovine serum sample. (a) Fluorescent images of beads from Cy5, TAMRA, and FAM. In 10% FBS, 250 nM FAM-PB-DNA A and FAM-NB-DNA B were incubated with H_2_O_2_ (0 or 1 mM), NR (0 or 250 μg/mL), and NADH (0 or 0.01 mM) in phosphate buffer (250 mM, pH 7.4) for 1 h at room temperature. Thereafter, reaction solutions were incubated with beads labeled with the DNA cA and DNA cB for 1 h at 37 °C to allow their accumulation on the beads. (b) Fluorescence intensity ratios (FAM/Cy5 and TAMRA/Cy5) for each Product DNA obtained from the beads in (a). Data are reported as mean ± SD (*n* = 30). Scale bars: 20 μm.

Finally, as a proof of concept for the broader utility of this platform, we demonstrated its coupling with oxidase enzymes.^13^ We tested a glucose oxidase system, where glucose oxidation triggers H_2_O_2_ generation. As shown in Figure S10, a distinct fluorescence signal was observed in the presence of glucose compared to the control. Although this is a preliminary demonstration, it confirms that our platform can be successfully integrated with enzymatic cascades to detect nonreactive substrates, expanding its potential applications.

It is worth noting the distinct advantages of this platform compared to existing DNA-encoded systems.^29–32^ Unlike conventional DNA-encoded libraries (DELs) or NGS-based methods that primarily rely on affinity-based binding and require time-consuming sequencing processes, our approach focuses on detecting “chemical reactivity” through a reaction-to-accumulation mechanism. This allows for the functional analysis of reactive biomolecules beyond simple binding events. Furthermore, the use of fluorescence-encoded beads enables a sequencing-free readout using standard fluorescence microscopy or flow cytometry. This feature significantly reduces the cost and turnaround time compared with NGS-based assays, offering a more accessible variation of DNA-based multiplexing for routine laboratory use. Future optimization of the system could include DNA-based signal amplification strategies to further improve the sensitivity.

## Conclusion

In summary, we developed a modular and highly versatile platform for the multiplexed detection of reactive biomolecules such as ROS and enzymes. On the basis of chemoresponsive fluorescent probe DNAs and fluorescence-encoded beads, we successfully prepared a molecular system. We modulated the phosphate backbone of the DNA strand by reaction site for target reactive biomolecules to form Probe DNAs. Probe DNAs gave Product DNAs by a specific reaction with the targets. We also prepared DNA-labeled fluorescent beads that were encoded the target molecules. The beads captured the Product DNA, enabling easy, simultaneous, and multiplex detection of the target molecules. This approach effectively overcomes the limitations of spectral overlap inherent in conventional fluorescence-based multiplex detection. Through systematic studies using H_2_O_2_ and nitroreductase as model analytes, we demonstrated sequence-specific and highly specific detection. Notably, our method maintains robust detection capabilities even in complex biological matrices such as serum. Given its scalability, sequencing-free readout, and compatibility with various reactive biomolecule classes, this platform provides a powerful and practical alternative to existing Raman- and NGS-based multiplex assays. It is expected to offer a high-throughput and comprehensive analysis of diverse enzymes and ROS in living systems that can be utilized in next-generation diagnostics and precision medicine.

## Supplementary Material



## Notes

Figures S1–S10, Table S1, synthetic procedures, and experimental details (PDF)

## Abbreviations

ROS: reactive oxygen species
HPLC: high-performance liquid chromatography
H_2_O_2_: hydrogen peroxide
*T*_m_: melting temperature
