# Supplementary material for: Multiplexed Detection of Reactive Biomolecules via Chemoresponsive DNA Accumulation on Fluorescence-Encoded Beads
Source: Chem Biomed Imaging. 2026 Jan 8;4(6):1062–7. doi: 10.1021/cbmi.5c00226 (PMC13291999; doi:10.1021/cbmi.5c00226)
Supplement: Supplementary file 1 [file im5c00226_si_001.pdf]

Supporting Information

Multiplexed Detection of Reactive Biomolecules via Chemo-responsive DNA Accumulation on Fluorescence-encoded Beads

Tatsuya Nishihara*[,1], Masato Sugawara[1], Reoto Mio[1], Yuto Motohashi, Kazuhito Tanabe*[, 1]

[1] Department of Chemistry and Biological Science, College of Science and Engineering, Aoyama Gakuin University, 5-10-1 Fuchinobe, Chuo-ku, Sagamihara, 252-5258, Japan

*Correspondence author: nishihara@chem.aoyama.ac.jp, tanabe.kazuhito@chem.aoyama.ac.jp

Table of Contents

1. Supporting Methods ········· **S4**

1-1. Synthesis ········· **S4**

1-2. Reaction monitoring of FAM-PB-DNA A and FAM-NB-DNA B ········· **S5**

1-3. CLSM fluorescence imaging of the DNA conjugated beads ········· **S7**

1-4. UV melting experiments ········· **S12**

2. Supporting Figures ········· **S13**

Figure S1. Synthesis of FAM-PB-DNA and FAM-NB-DNA ········· **S13**

Figure S2. Evaluation of accumulation of FAM-DNA probe and FAM product DNA to the beads ········· **S14**

Figure S3. Reaction monitoring of FAM-PB-DNA and FAM-NB-DNA ········· **S15**

Figure S4. Reactivity of FAM-PB-DNA with various ROS ········· **S16**

Figure S5. Concentration-dependent responses of FAM-PB-DNA to H_2O_2 and FAM-NB-DNA to NR ········· **S17**

Figure S6. Evaluation of potential spectral crosstalk in the three-color detection system ········· **S18**

Figure S7. Preparation of fluorescence-encoded beads ········· **S19**

Figure S8. Simultaneous detection of product DNA cocktail ········· **S21**

Figure S9. Simultaneous detection of H_2O_2 and NR using DNA probe cocktail ························ S22

Figure S10. Application of FAM-PB-DNA for glucose detection coupled with glucose oxidase ························ S23

Table S1. DNA sequence in this study ························ S24

Experimental section

1. Supporting Methods

General Methods

Reagents were purchased from Wako pure chemical industries, Sigma Aldrich, Tokyo chemical industries, micromod Partikeltechonogie GmbH, and eurofins Genomics.

Analytical and preparative high-performance liquid chromatographies were performed on a D2000 HPLC system and L7000 HPLC system (HITACHI) with reversed phase column (InerrtsilvODS-3, GL Science Inc., ϕ 1.0 mm×250 mm (particle size; 5 μm)).

Beads observations were used by a C2 confocal microscope (NIKON). UV melting experiments were obtained at 260 nm using a JASCO V-630 UV/VIS spectrophotometer. MALDI-TOF mass spectrometry were recorded on an MALDI-8030 (SHIMADZU).

1-1. Synthesis

Synthesis of FAM-PB-DNA A (Figure S1)

2-(4-Bromomethylphenyl)-4,4,5,5-tetramethyl-1,3,2-dioxaborolane (PB) (10 mmol) were added the solution of FAM-PS-DNA A (5.0 nmol) in 5 mM pH6.0 phosphate buffer and incubated at room temperature for 6 h. The reaction mixture was purified using Micro Bio-Spin™ 6 Columns (Bio-Rad) and milliQ water as an elution solution. The concentration of the oligomer was determined by complete digestion with alkaline phosphatase (AP), nuclease P1 (PI) and phosphodiesterase I at 37°C for overnight. Identities of synthesized oligomers were confirmed by MALDI-TOF MS spectrometry ($[M-H]^-$ calcd. 5855.0, found. 5855.2).

The purity of synthesized oligomer was confirmed by monitoring of UV absorbance at 494 nm at a flow rate of 0.6 (analysis) $mLmin^{-1}$ with a 0–25% (0–50 min) and 25–100% (50–60 min) and 100%

(60–70 min) gradient of ACN/TEAA buffer (100 mM, pH 7.0).

Synthesis of FAM-NB-DNA B (Figure S1)

4-nitrobenzyl bromide (NB) (10 mmol) were added the solution of FAM-PS-DNA B (5.0 nmol) in 5 mM pH6.0 Phosphate buffer and incubated at room temperature for 6 h. The reaction mixture was purified using Micro Bio-Spin™ 6 Columns (Bio-Rad) and milliQ water as an elution solution. The concentration of the oligomer was determined by complete digestion with alkaline phosphatase (AP), nuclease P1 (PI) and phosphodiesterase I at 37°C for overnight. Identities of synthesized oligomers were confirmed by MALDI-TOF MS spectrometry ($[M-H]^-$ calcd. 5612.1, found. 5612.3).

The purity of synthesized oligomer was confirmed by monitoring of UV absorbance at 494 nm at a flow rate of 0.6 (analysis) $mLmin^{-1}$ with a 0–25% (0–50 min) and 25–100% (50–60 min) and 100% (60–70 min) gradient of ACN/TEAA buffer (100 mM, pH 7.0).

1-2. Reaction monitoring of FAM-PB-DNA A and FAM-NB-DNA B

The reaction mixtures were analyzed by HPLC. The column eluents were monitored by the UV absorbance at 494 nm at a flow rate of 0.6 (analysis) $mLmin^{-1}$ with a 0–25% (0–50 min) and 25–100% (50–60 min) and 100% (60–70 min) gradient of ACN/TEAA buffer (100 mM, pH 7.0).

Reaction monitoring of FAM-PB-DNA A (Figure S3)

5 μM FAM-PB-DNA A was reacted with 0, 0.1, 0.3, 0.5, or 1 mM H_2O_2 in 250 mM phosphate buffer (pH 7.4) for 1 h at room temperature.

The reaction mixture with H_2O_2 was mixed (20 μL) with 50 μM FAM-product DNA A (FAM-PS-DNA A) (2 μL, 100 pmol). These samples were analyzed by HPLC as described above.

Reactivity of FAM-PB-DNA with various ROS (Figure S4)

5 μM FAM-PB-DNA A was reacted with 0 or 1 mM H_2O_2, potassium superoxide, NaOCl, NOC7 or AAPH in PB (250 mM, pH 7.4) for 1 h at room temperature. The reaction mixtures were analyzed by HPLC as described above.

Sample preparation

H_2O_2. The H_2O_2 solution was diluted with milliQ water.

ROO˙. 2,2´-Azobis(2-amidinopropane)dihydrochloride (AAPH) was dissolved in milliQ water. The solution was mixed with the FAM-PB-DNA A solution immediately.

O_2˙-. Potassium superoxide was dissolved in milliQ water. The solution was mixed with the FAM-PB-DNA A solution immediately.

NO. 1-Hydroxy-2-oxo-3-(N-methyl-3-aminopropyl)-3-methyl-1-triazone (NOC7) was dissolved in milliQ solution. The solution was mixed with the FAM-PB-DNA A solution immediately.

-OCl. NaOCl solution was diluted with milliQ solution.

Reaction monitoring of FAM-NB-DNA B (Figure S3)

5 μM FAM-NB-DNA B was reacted with 0, 75, 125, or 250 μg/mL nitroreductase (NR), recombinant from *E. coli.* (Sigma Aldrich, N9284) and 0 or 1 mM NADH in 250 mM phosphate buffer (pH 7.4) for 1 h at room temperature. The reaction mixture with NR was mixed (20 μL) with 50 μM FAM-product DNA B (FAM-PS-DNA B) (2 μL, 100 pmol). These samples were analyzed by HPLC as described above.

1-3. CLSM fluorescence imaging of the DNA conjugated beads

Annealing condition

The DNA solution was heated 90˚C for 5 min and then cooled at room temperature.

Preparation of beads

25 mg/mL streptavidin coated beads (2 μL/ sample, 01-19-403_size 4 μm or 01-19-503_size 5 μm or 01-19-603_size 6 μm, micromod Partikeltechonogie GmbH) was centrifuged for 20 min at 15,000 g at 25°C, and supernatant was removed. Binding buffer (10 μL/sample, 1 M NaCl, 500 μM EDTA, 5 mM Tris-HCl pH 7.4) was added to the beads for washing. The suspension solution was centrifuged for 20 min at 15,000 g at 25°C, and supernatant was removed. The washing procedure performed again. Binding buffer (10 μL/sample) was added to the beads.

DNA labelling to the streptavidin coated beads

DNA complex tethered with biotin (1 pmol/sample) and streptavidin coated beads was mixed in the binding buffer over night at 4°C (50 μL/sample). The suspension solution was centrifuged for 5 min at 15,000 g at 4°C, and supernatant was removed. This washing procedure performed three times by using the 10 mM phosphate buffer (pH 7.4) with 100 mM NaCl. After washing the beads, sample was incubated with the Template DNA labeled beads in appropriate buffer for 1 h at 37°C to accumulate on the beads. The beads solution was observed by CLSM. Excitation and emission wavelengths were as follow: Cy5: λ_{ex} = 640 nm, λ_{em} = 640–1000 nm, TAMRA: λ_{ex} = 561 nm, λ_{em} 570–1000 nm, FAM: λ_{ex} = 488 nm, λ_{em} = 500–550 nm.

Temperature dependency of accumulation of FAM-DNA probes and FAM-product DNA (FAM-PS-DNA) to the beads (Figure S2)

DNA labelling to the streptavidin coated beads

10 pmol Template DNA cA was annealed with 1 pmol Cy5-DNA-Biotin in 5 mM $MgCl_2$ aq. DNA labelling to the streptavidin coated beads and washing was performed as described above. After washing the beads, 5 pmol FAM-product DNA A (FAM-PS-DNA A), FAM-PB-DNA A or FAM-NB-DNA A were incubated with the Template DNA cA labeled beads in the 10 mM phosphate buffer (pH 7.4) with 100 mM NaCl for 1 h at 4°C, room temperature, or 37°C to accumulate on the beads. The beads solution was observed by CLSM as described above.

Dose dependency of FAM-PS-DNA for the accumulation on the beads (Figure S2)

10 pmol Template DNA cA was annealed with 1 pmol Cy5-DNA-Biotin and 10 pmol TAMRA-DNA 1 in 5 mM $MgCl_2$ aq. DNA labelling to the streptavidin coated beads and washing was performed as described above. After washing the beads, 0, 1, 5, or 10 pmol FAM-PS-DNA A was incubated with the Template DNA cA labeled beads in the 10 mM phosphate buffer (pH 7.4) with 100 mM NaCl for 1 h at 37°C to accumulate on the beads. The beads solution was observed by CLSM as described above.

Detection of H_2O_2 using DNA labeled beads and FAM-PB-DNA A (Figure 3, Figure S5)

H_2O_2 reaction solution: 250 nM FAM-PB-DNA A or FAM-product DNA A (FAM-PS-DNA A) was reacted with 0, 0.03, 0.1, 0.3 or 1 mM H_2O_2 in phosphate buffer (250 mM, pH 7.4) for 1 h at room temperature.

10 pmol Template DNA cA was annealed with 1 pmol Cy5-DNA-Biotin and 10 pmol TAMRA-DNA 1 in 5 mM $MgCl_2$ aq. DNA labelling to the streptavidin coated beads and washing was performed as

described above. After washing the beads, H_2O_2 reaction solution (20 μL) and 10 mM phosphate buffer (pH 7.4) with 100 mM NaCl (80 μL) was incubated with the Template DNA cA labeled beads for 1 h at 37°C to accumulate on the beads. The beads solution was observed by CLSM as described above.

Detection of NR using DNA labeled beads and FAM-NB-DNA B (Figure 3, Figure S5)

NR reaction solution: 250 μM FAM-NB-DNA B or FAM-product DNA B (FAM-PS-DNA B) was reacted with 0, 0.75, 25, 75, 250 or 500 μg/mL NR and 0, 0.01 or 1 mM NADH in phosphate buffer (250 mM, pH 7.4) for 1 h at room temperature.

10 pmol Template DNA cB was annealed with 1 pmol Cy5-DNA-Biotin and 10 pmol TAMRA-DNA 1 in 5 mM $MgCl_2$ aq. DNA labelling to the streptavidin coated beads and washing was performed as described above. After washing the beads, NR reaction solution (20 μL) and 10 mM phosphate buffer (pH 7.4) with 100 mM NaCl (80 μL) was incubated with the Template DNA cB labeled beads for 1 h at 37°C to accumulate on the beads. The beads solution was observed by CLSM as described above.

Detection of glucose using DNA labeled beads and FAM-PB-DNA coupled with glucose oxidase (GOx) (Figure S10)

GOx reaction solution: 250 nM FAM-PB-DNA A or FAM-product DNA A (FAM-PS-DNA A) was reacted with 0 or 7.5 mM glucose and 1.0 U/μL GOx in phosphate buffer (250 mM, pH 7.4) for 1 h at 37°C.

10 pmol Template DNA cA was annealed with 1 pmol Cy5-DNA-Biotin and 10 pmol TAMRA-DNA 1 in 5 mM $MgCl_2$ aq. DNA labelling to the streptavidin coated beads and washing was performed as described above. After washing the beads, GOx reaction solution (20 μL) and 10 mM phosphate buffer (pH 7.4) with 100 mM NaCl (80 μL) was incubated with the Template DNA cA labeled beads for 1 h

at 37°C to accumulate on the beads. The beads solution was observed by CLSM as described above.

Fluorescence measurement of beads with different fluorescence intensity ratios by Cy5 and TAMRA (Figure S7 a,b)

Preparation of DNA complexes

DNA solution a: 10 pmol Template DNA cA was annealed with 1 pmol Cy5-DNA-Biotin and 10 pmol TAMRA-DNA 1 in 5 mM $MgCl_2$ aq.

DNA solution b: 10 pmol Template DNA cA was annealed with 1 pmol Cy5-DNA-Biotin and 10 pmol DNA 1 in 5 mM $MgCl_2$ aq.

DNA solution a and **DNA solution b** was mixed with each other (9:1, 5:5, 3:7 or 1:9).

Figure S7 a

DNA labelling to the streptavidin coated beads (4 μm) and washing was performed as described above. After washing the beads, 5 pmol FAM-product DNA A (FAM-PS-DNA A) was incubated with the Template DNA cA labeled beads in the 10 mM phosphate buffer (pH 7.4) with 100 mM NaCl for 1 h at 37°C to accumulate on the beads. The beads solution was observed by CLSM as described above.

Figure S7 b

DNA labelling to the streptavidin coated beads (6 μm) and washing was performed as described above. After washing the beads, the beads in the 10 mM phosphate buffer (pH 7.4) with 100 mM NaCl solution was observed by CLSM as described above.

Fluorescence measurement of beads of different sizes (Figure S7 c)

10 pmol Template DNA cA was annealed with 1 pmol Cy5-DNA-Biotin in 5 mM $MgCl_2$ aq. DNA

labelling to the streptavidin coated beads (4, 5, or 6 μm) and washing was performed as described above. After washing the beads, each sizes beads were mixed in the 10 mM phosphate buffer (pH 7.4) with 100 mM NaCl for 1 h at 37°C. The beads solution was observed by CLSM as described above.

Sequence-specific DNA accumulation on beads (Figure S8)

Preparation of DNA complex: TAMRA high (DNA A accumulation)

DNA solution a: 10 pmol Template DNA cA was annealed with 1 pmol Cy5-DNA-Biotin and 10 pmol TAMRA-DNA 1 in 5 mM $MgCl_2$ aq.

DNA solution b: 10 pmol Template DNA cA was annealed with 1 pmol Cy5-DNA-Biotin and 10 pmol DNA 1 in 5 mM $MgCl_2$ aq.

DNA solution a and **DNA solution b** was mixed with each other (9:1).

Preparation of DNA complex: TAMRA low (DNA B accumulation)

DNA solution c: 10 pmol Template DNA cB was annealed with 1 pmol Cy5-DNA-Biotin and 10 pmol TAMRA-DNA 1 in 5 mM $MgCl_2$ aq.

DNA solution d: 10 pmol Template DNA cB was annealed with 1 pmol Cy5-DNA-Biotin and 10 pmol DNA 1 in 5 mM $MgCl_2$ aq.

DNA solution c and **DNA solution d** was mixed with each other (3:7).

DNA labelling to the streptavidin coated beads and washing was performed respectively as described above. After washing the beads, 10 mM phosphate buffer (pH 7.4) with 100 mM NaCl (20 μL) was added respectively and mixed with each other to prepare the beads cocktail, which shows different fluorescent ratio (TAMRA/Cy5). 0 or 5 pmol FAM-PS-DNA A and FAM-PS-DNA B were incubated

with the Template DNA cA and DNA cB labeled beads cocktail in the 10 mM phosphate buffer (pH 7.4) with 100 mM NaCl for 1 h at 37°C to accumulate on the beads. The beads solution was observed by CLSM as described above.

Simultaneous detection of H_2O_2 and NR using the DNA probe cocktail (Figure 4, S9)

Template DNA labelled beads cocktail was prepared as described above (page S11).

250 nM FAM-PB-DNA A and 250 nM FAM-NB-DNA B were incubated with H_2O_2 (0 or 1 mM), NR (0 or 250 µg/mL) with NADH (0 or 0.01 mM) in phosphate buffer (250 mM, pH 7.4) with or without 10% fetal bovine serum (FBS) for 1 h at room temperature. After reaction solutions were incubated with the Template DNA cA and DNA cB labeled beads cocktail for 1 h at 37°C to accumulate on the beads. The beads solution was observed by CLSM as described above.

1-4. UV melting experiments (Figure 2)

DNA probe and complementary DNA or DNA product and complementary DNA were dissolved 100 mM NaCl to give a final strand concentration of 4.0 µM in advance to the UV melting experiments.

2. Supporting Figures

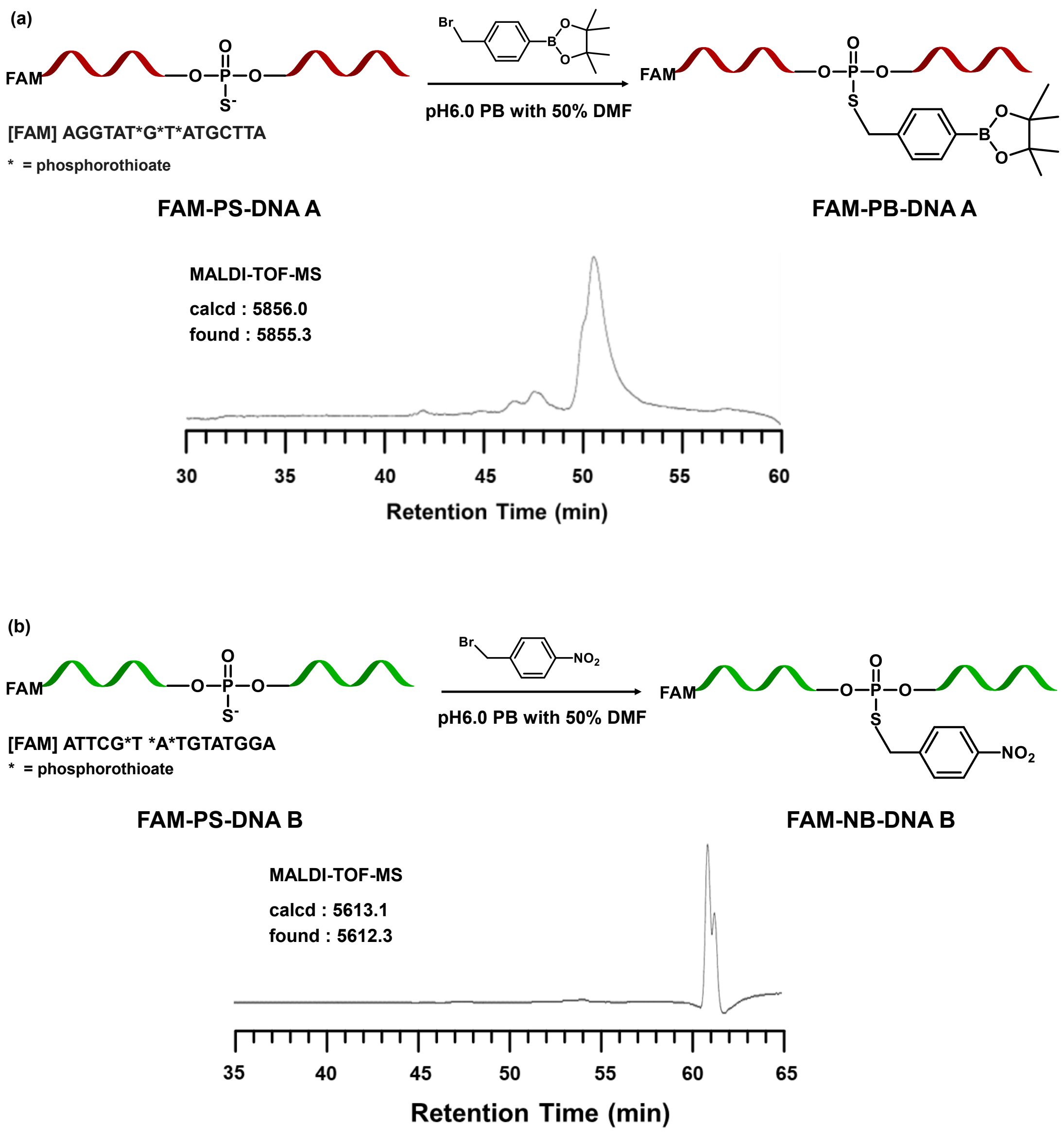

Figure S1. (a) Synthesis of FAM-PB-DNA A. HPLC chart of FAM-PB-DNA A. (b) Synthesis of FAM-NB-DNA B. HPLC chart of FAM-NB-DNA B.

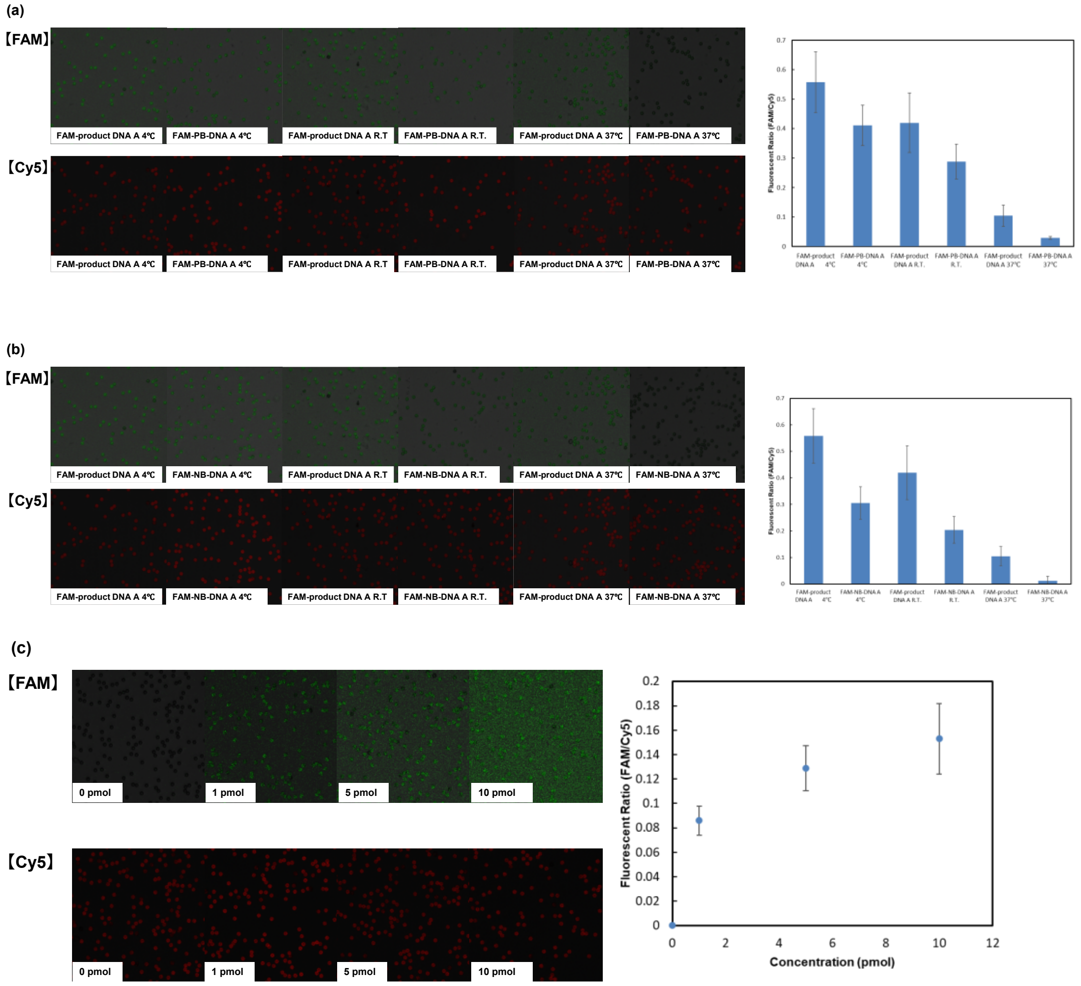

Figure S2. (a) Fluorescent images of bead from FAM and Cy5. 5 pmol FAM-product DNA A (FAM-PS-DNA) or FAM-PB-DNA A was incubated with the DNA cA labeled beads for 1 h at 4°C, room temperature or 37°C to accumulate on the beads. Fluorescence intensity ratio (FAM/Cy5) obtained from the beads. Data = Mean ± SD (n = 30). (b) Fluorescent images of bead from FAM and Cy5. 5 pmol FAM-PS-DNA A or FAM-NB-DNA A was incubated with the DNA cA labeled beads for 1 h at 4°C, room temperature or 37°C to accumulate on the beads. Fluorescence intensity ratio (FAM/Cy5) obtained from the beads. Data = Mean ± SD (n = 30). (c) Fluorescent images of bead from FAM and Cy5. 0, 1, 5, or 10 pmol FAM-PS-DNA A was incubated with the DNA cA labeled beads for 1 h at 37°C to accumulate on the beads. Fluorescence intensity ratio (FAM/Cy5) obtained from the beads. Data = Mean ± SD (n = 30).

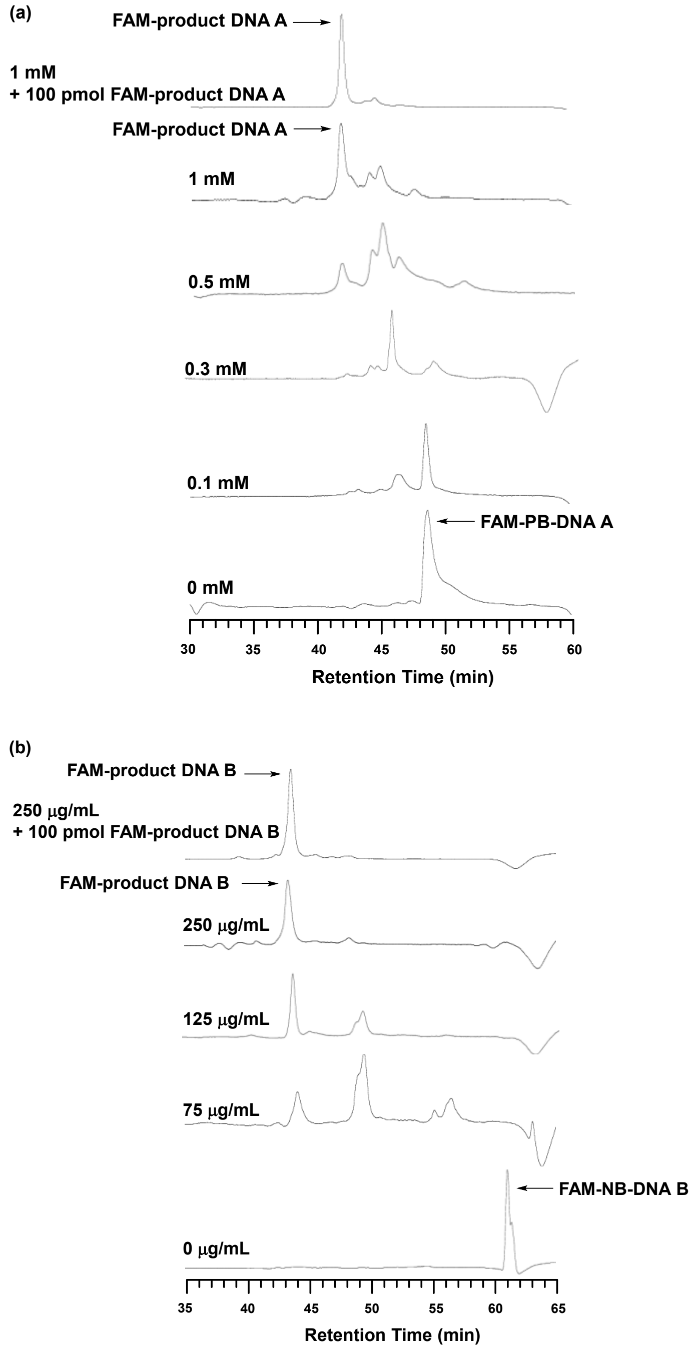

Figure S3. (a) Reaction monitoring using HPLC (λ= 494 nm); 5 µM FAM-PB-DNA A reacted with 0, 0.1, 0.3, 0.5 or 1 mM H_2O_2 in 250 mM phosphate buffer (pH 7.4) for 1 h at room temperature. (b) Reaction monitoring using HPLC (λ= 494 nm); 5 µM FAM-NB-DNA B reacted with 0, 75, 125, or 250 µg/mL NR and 1 mM NADH in 250 mM phosphate buffer (pH 7.4) for 1 h at room temperature.

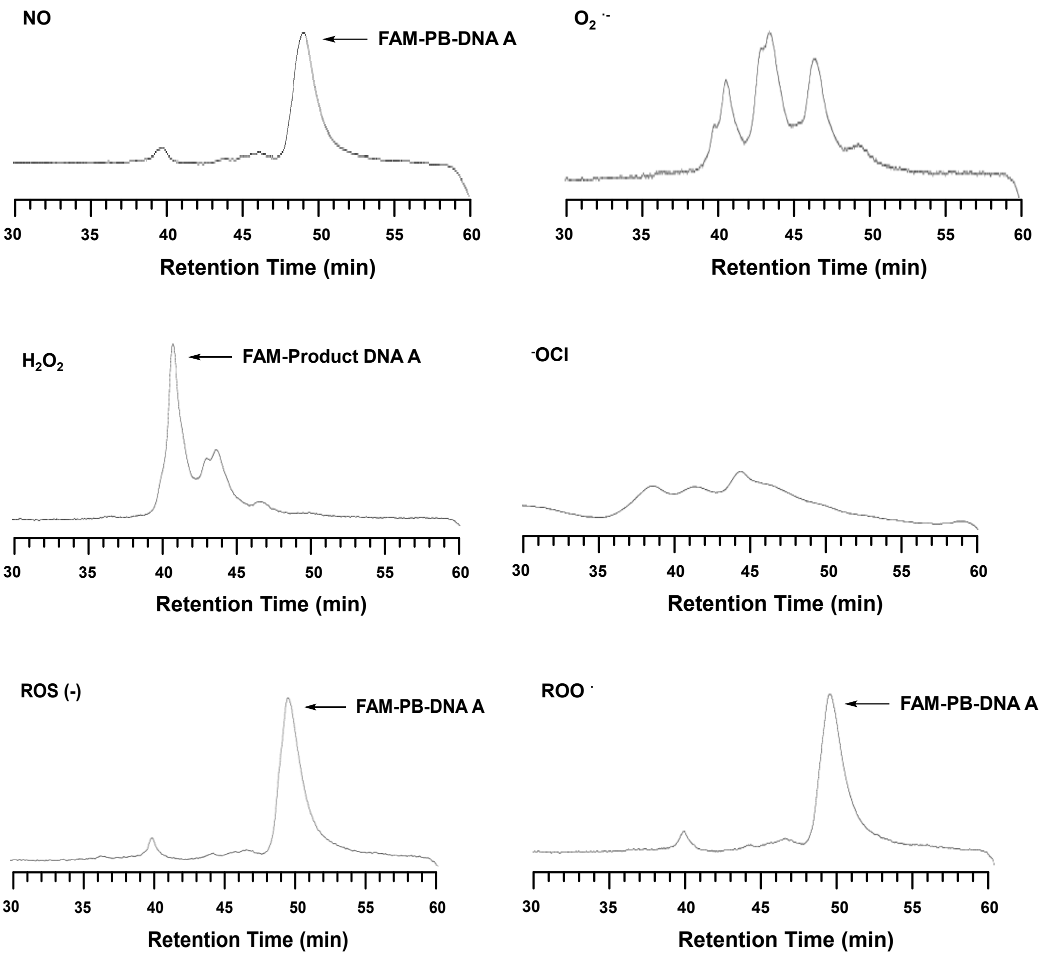

Figure S4. Reactivity of FAM-PB-DNA with various ROS. Reaction monitoring using HPLC (λ= 494 nm); 5 μM FAM-PB-DNA A reacted with 1 mM H_2O_2, potassium superoxide, NaOCl, NOC7 or AAPH in PB (250 mM, pH 7.4) for 1 h at room temperature.

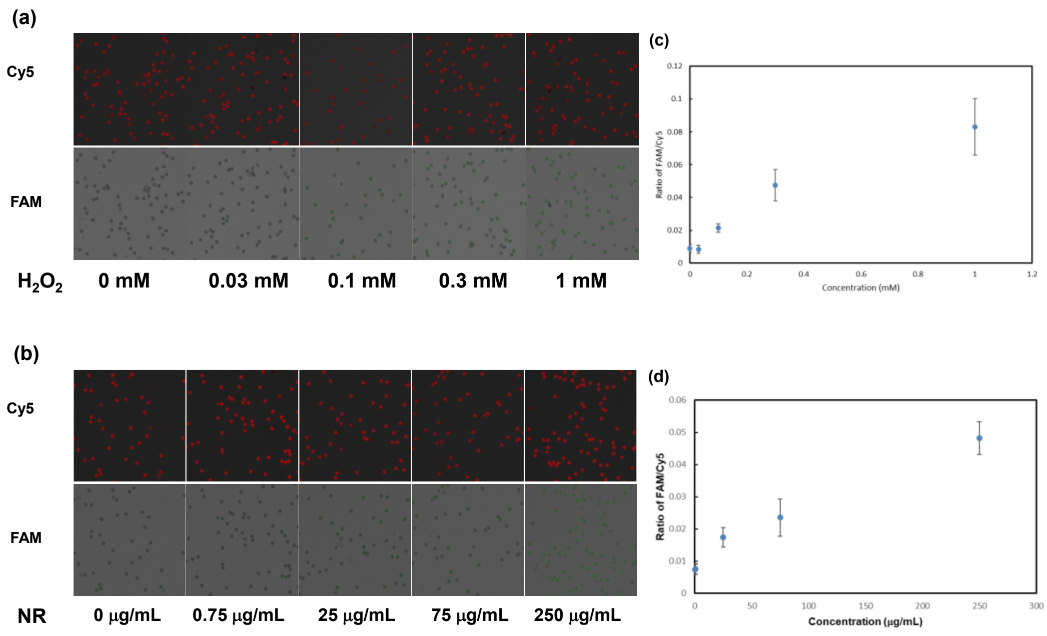

Figure S5. (a) Fluorescent images of bead from FAM, Cy5. 250 nM FAM-PB-DNA A that were allowed to react with 0, 0.03, 0.1, 0.3 or 1 mM H_2O_2 in 250 mM phosphate buffer (pH 7.4) for 1 h at room temperature. (b) Fluorescent images of bead from FAM, Cy5. 250 nM FAM-NB-DNA B that were allowed to react with 0, 0.75, 25, 75 or 250 µg/mL NR and 0.01 mM NADH in 250 mM phosphate buffer (pH 7.4) for 1 h at 37°C. After washing the beads solution, the reaction solutions were incubated with the cDNA labeled beads for 1 h at 37°C to accumulate on the beads. (c,d) Fluorescence intensity ratio (FAM/Cy5) obtained from the beads in Figure a, b. Data = Mean ± SD (n = 30).

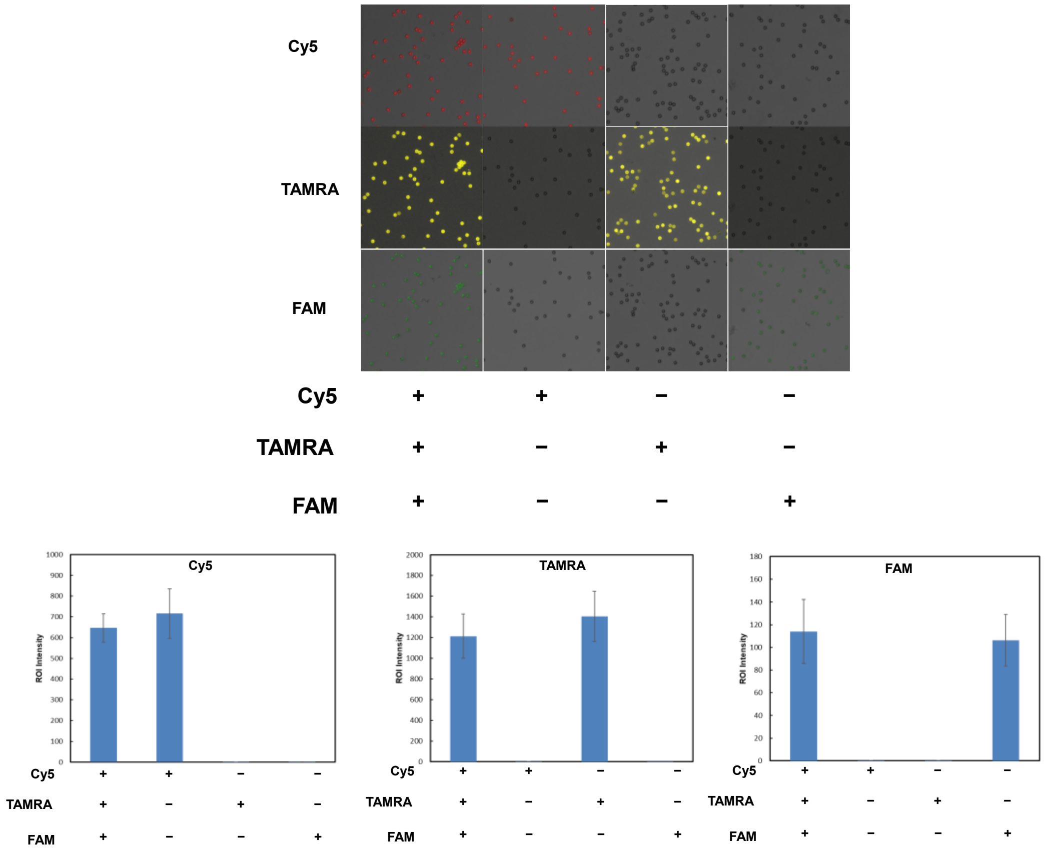

Figure S6. Comparison of fluorescence signals between single-color and three-color encoded beads. The fluorescence signals of beads containing only a single dye were compared with those of beads containing all three dyes. Fluorescence intensity obtained from the beads. Data = Mean ± SD (n = 30). The overlap indicates negligible spectral interference among the dyes under our experimental conditions.

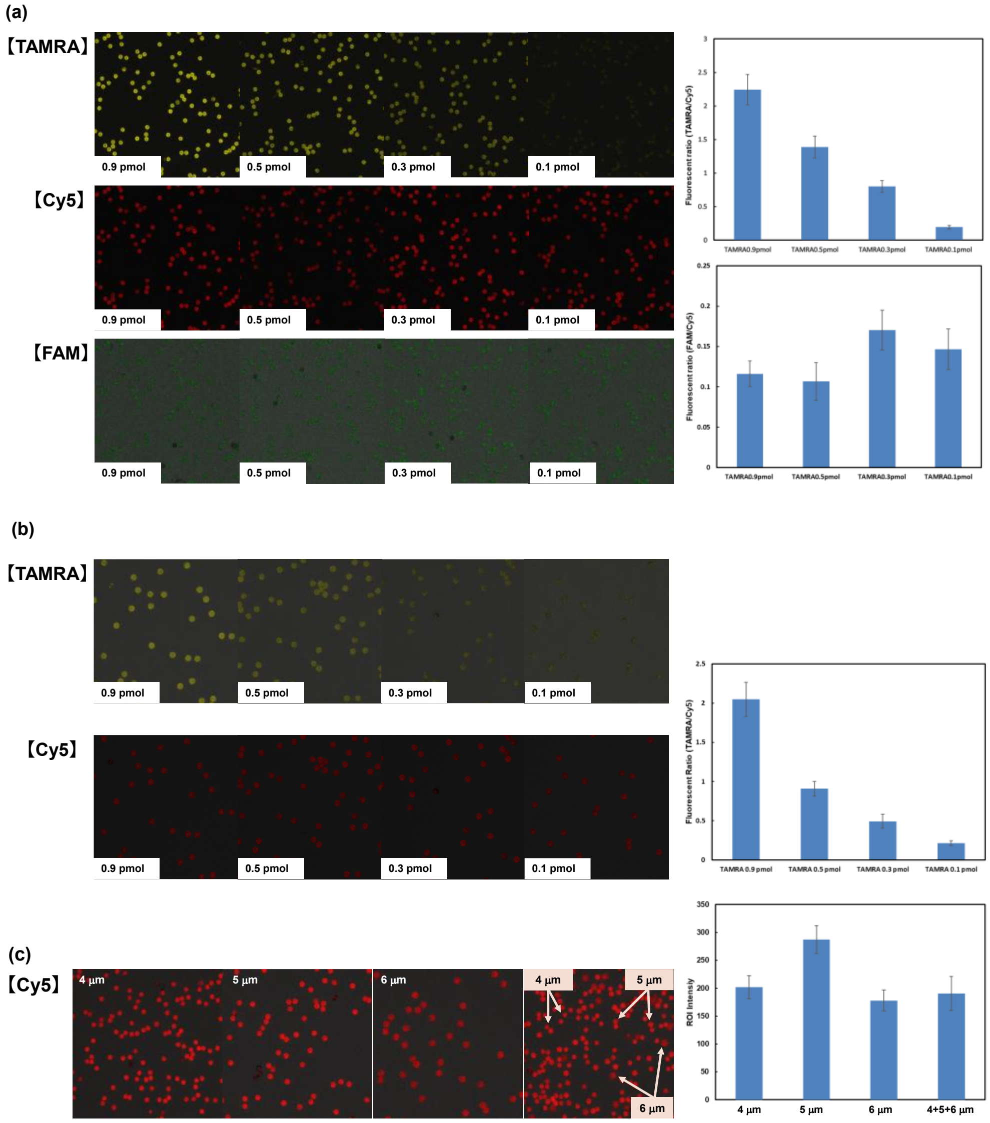

Figure S7. (a) Fluorescent images of bead from FAM, Cy5 and TAMRA. 10 pmol Template-DNA cA was annealed with 1 pmol Cy5-DNA-Biotin and 0.9 or 0.5 or 0.3 or 0.1 pmol TAMRA-DNA 1. The DNA complex was labelled with the beads (4 µm) in 10 mM phosphate-buffered saline for overnight at 4°C. After washing the solution, 5 pmol FAM-PS-DNA A was incubated with the DNA cA labeled beads for 1 h at 37°C to accumulate on the beads. Fluorescence intensity ratio (TAMRA/Cy5 and FAM/Cy5) obtained from the beads. Data = Mean ± SD (n = 30). (b) Fluorescent images of bead from Cy5 and TAMRA. 10 pmol Template-DNA cA was annealed with 1 pmol Cy5-DNA-Biotin and 0.9 or 0.5 or 0.3 or 0.1 pmol TAMRA-DNA 1. The DNA complex was labelled with the beads (6 µm) in 10 mM phosphate-buffered saline for overnight at 4°C. After washing the solution, 5 pmol FAM-product DNA A was incubated with the DNA cA labeled beads for 1 h at 37°C to accumulate on the beads. Fluorescence intensity ratio (TAMRA/Cy5) obtained

from the beads. Data = Mean ± SD (n = 22). (c) Fluorescent images of bead from Cy5. 10 pmol Template-DNA cA was annealed with 1 pmol Cy5-DNA-Biotin. The DNA complex was labelled with the beads (4, 5, or 6 μm) in 10 mM phosphate-buffered saline for overnight at 4°C. Fluorescence intensity (Cy5) obtained from the beads. Data = Mean ± SD (n = 15).

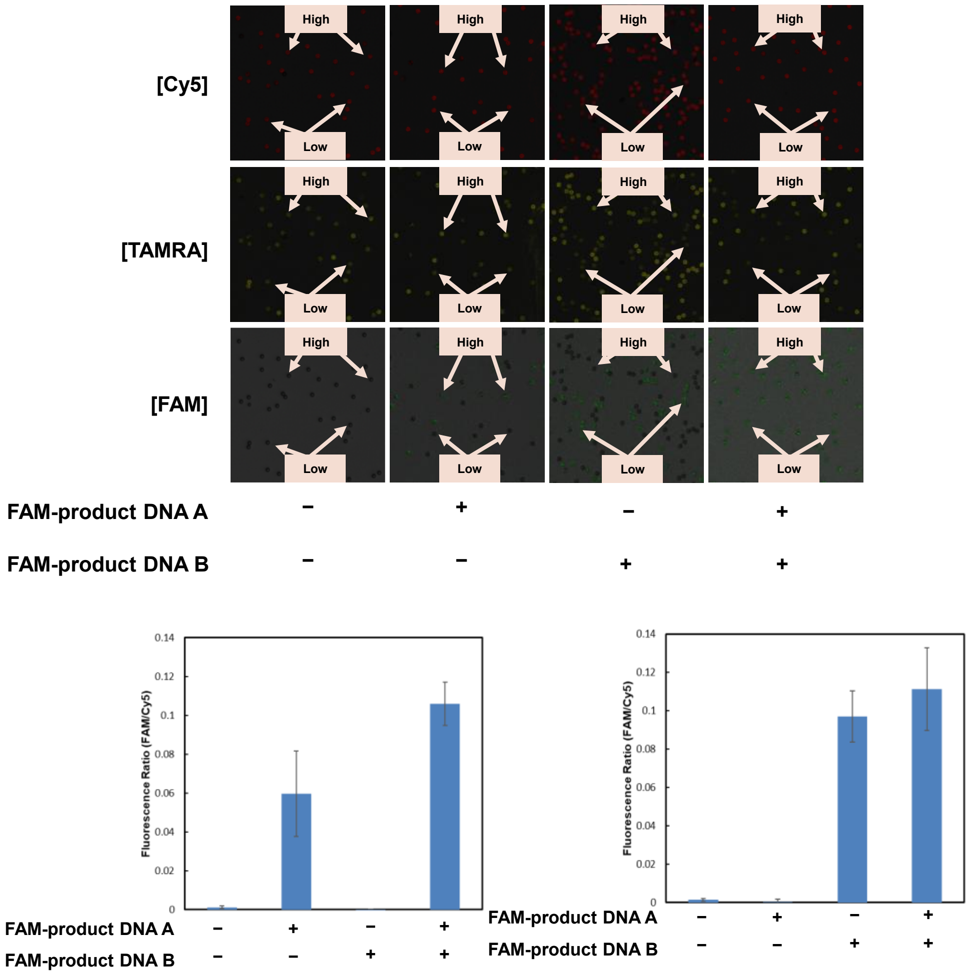

Figure S8. Fluorescent images of bead from FAM, Cy5 and TAMRA. 10 pmol Template-DNA cA and Template-DNA cB were annealed with 1 pmol Cy5-DNA-Biotin and 0.9 or 0.1 pmol TAMRA-DNA 1. The DNA complex was labelled with the beads (4 µm) in 10 mM phosphate-buffered saline for overnight at 4°C, respectively. After washing the solution, 0 or 5 pmol FAM-PS-DNA A and FAM-PS-DNA B were incubated with mixture of DNA cA and DNA cB labeled beads for 1 h at 37°C to accumulate on the beads. Fluorescence intensity ratio (FAM/Cy5) obtained from the beads. Data = Mean ± SD (n = 10).

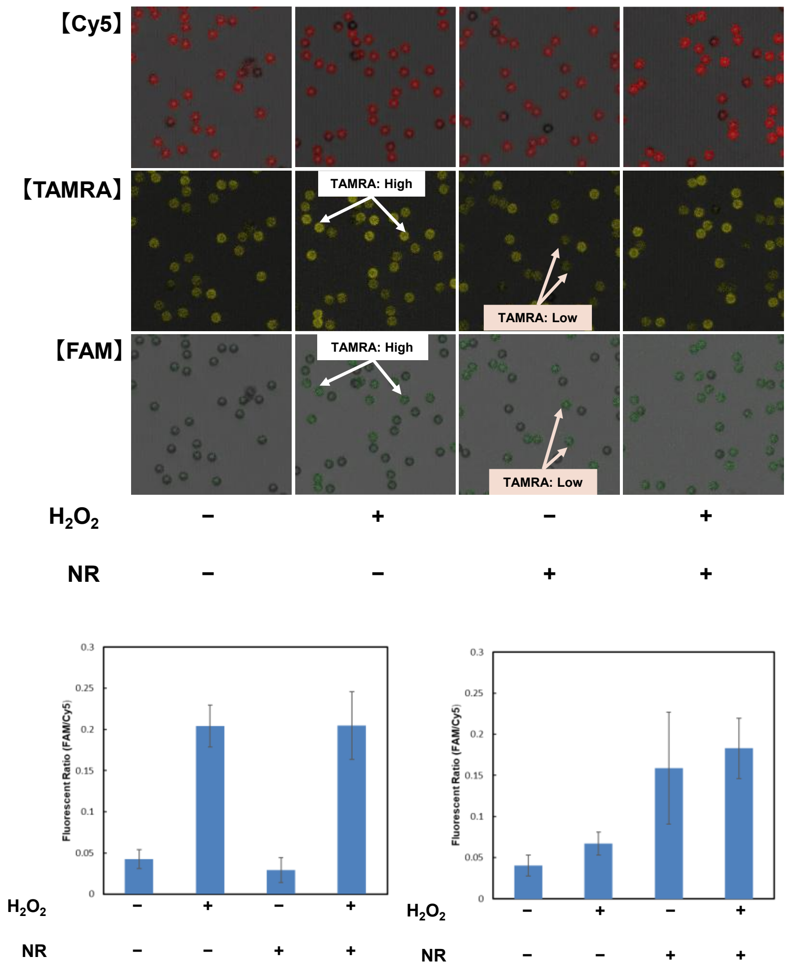

Figure S9. Fluorescent images of bead from FAM, Cy5 and TAMRA. 250 nM FAM-PB-DNA A and 250 nM FAM-NB-DNA B were incubated with H_2O_2 (0 or 1 mM) NR (0 or 250 μg/mL) and NADH (0 or 0.01 mM) in phosphate buffer (250 mM, pH 7.4) for 1 h at room temperature. After reaction solutions were incubated with the DNA cA and DNA cB labeled beads for 1 h at 37°C to accumulate on the beads. Fluorescence intensity ratio (FAM/Cy5) for each product DNA obtained from the beads. Data = Mean ± SD (n = 15).

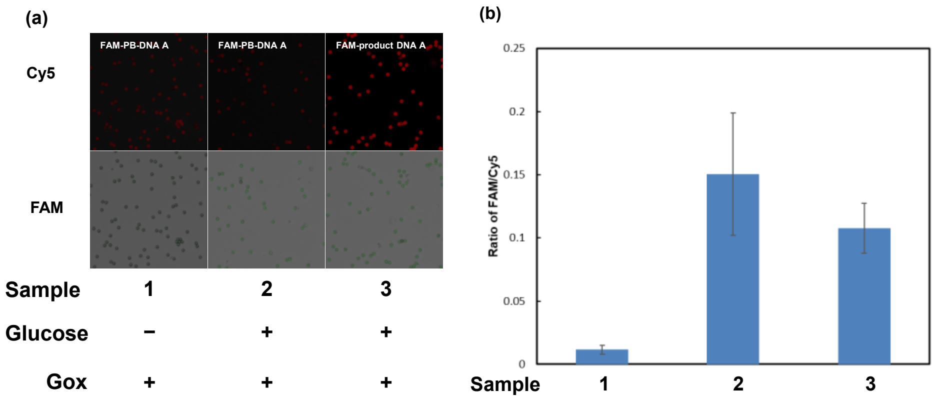

Figure S10. Detection of glucose using DNA labeled beads and FAM-PB-DNA coupled with GOx. (a) Fluorescent images of bead from Cy5 and FAM. 250 nM FAM-PB-DNA A were incubated with Glucose (0 or 7.5 mM), GOx (1 U/μL) in phosphate buffer (250 mM, pH 7.4) for 1 h at 37°C. After reaction solutions were incubated with the DNA cA labeled beads for 1 h at 37℃ to accumulate on the beads. Fluorescence intensity ratio (FAM/Cy5) for each product DNA obtained from the beads. Data = Mean ± SD (n = 30).

Table S1. DNA Sequences used in this study

Strand Name	Sequence data (5'→3') * = Phosphorothioate
FAM-product DNA A (FAM-PS-DNA A)	[FAM]AGG TAT*G*T*A TGC TTA
FAM-product DNA B (FAM-PS-DNA B)	[FAM]ATT CG*T*A*TG TAT GGA
Template-DNA cA	GAA TTC CTG TAG ATT GGA CCA AGC TTC GAG TGA CTG CGA TGA CCC TAA GCA TAC ATA CCT
Template-DNA cB	GAA TTC CTG TAG ATT GGA CCA AGC TTC GAG TGA CTG CGA TTC CAT ACA TAC GAA TCC CAG
Cy5-DNA-biotin	[Cy5]GGT CCA ATC TAC AGG AAT TC[BIOTEG]
TAMRA-DNA 1	[TAM] ATC GCA GTC ACT CGA AGC TT
DNA 1	ATC GCA GTC ACT CGA AGC TT
FAM-PS(19)-DNA	[FAM] AGG TAT GTA TGC TTA GGG T*C
FAM-PS(10)-DNA	[FAM] AGG TAT GTA T*GC TTA GGG TC
FAM-PS-(18.19)-DNA	[FAM] AGG TAT GTA TGC TTA GGG *T*C
FAM-PS-(10.19)-DNA	[FAM] AGG TAT GTA T*GC TTA GGG T*C
FAM-PS-(10.11)-DNA	[FAM] AGG TAT GTA T*G*C TTA GGG TC
FAM-PS-(5.10.15)-DNA	[FAM] AGG TA*T GTA T*GC TTA *GGG TC
FAM-PS-(9.10.11)-DNA	[FAM] AGGTATGTA *T*G*C TTAGGGTC
